# Cognitive impairment influences the risk of reoperation after hip fracture surgery: results of 87,573 operations reported to the Norwegian Hip Fracture Register

**DOI:** 10.1080/17453674.2019.1709712

**Published:** 2020-01-13

**Authors:** Målfrid Holen Kristoffersen, Eva Dybvik, Ole Martin Steihaug, Torbjørn Berge Kristensen, Lars Birger Engesaeter, Anette Hylen Ranhoff, Jan-Erik Gjertsen

**Affiliations:** a Norwegian Hip Fracture Register, Department of Orthopedic Surgery, Haukeland University Hospital, Bergen;; b Department of Clinical Medicine, Faculty of Medicine, University of Bergen, Bergen;; c Emergency Department, Haukeland University Hospital, Bergen;; d Diakonhjemmet Hospital, Oslo;; e Department of Clinical Sciences, Faculty of Medicine, University of Bergen, Bergen, Norway

## Abstract

Background and purpose — About one-fourth of hip fracture patients have cognitive impairment. We investigated whether patients’ cognitive function affects surgical treatment, risk of reoperation, and mortality after hip fracture, based on data in the Norwegian Hip Fracture Register (NHFR).

Patients and methods — This prospective cohort study included 87,573 hip fractures reported to the NHFR in 2005–2017. Hazard rate ratios (HRRs) for risk of reoperation and mortality were calculated using Cox regression adjusted for sex, age, ASA class, fracture type, and surgical method.

Results — Cognitive impairment was reported in 27% of patients. They were older (86 vs. 82 years) and had higher ASA class than non-impaired patients. There were no differences in fracture type or operation methods. Cognitively impaired patients had a lower overall reoperation rate (4.7% vs. 8.9%, HRR 0.71; 95% CI 0.66–0.76) and lower risk of reoperation after osteosynthesis (HRR 0.58; CI 0.53–0.63) than non-impaired patients. Cognitively impaired hip fracture patients had an increased reoperation risk after hemiarthroplasty (HRR 1.2; CI 1.1–1.4), mainly due to dislocations (1.5% vs. 1.0%, HRR 1.7; CI 1.3–2.1). Risk of dislocation was particularly high following the posterior approach (4.7% vs. 2.8%, HRR 1.8; CI 1.2–2.7). Further, they had a higher risk of reoperation due to periprosthetic fracture after uncemented hemiarthroplasty (HRR 1.6; CI 1.0–2.6). Cognitively impaired hip fracture patients had higher 1-year mortality than those without cognitive impairment (38% vs. 16%, HRR 2.1; CI 2.1–2.2).

Interpretation — Our findings support giving cognitively impaired patients the same surgical treatment as non-impaired patients. But since the risk of hemiprosthesis dislocation and periprosthetic fracture was higher in cognitively impaired patients, they should probably not have posterior approach surgery or uncemented implants.

In Norway, with a population of 5.2 million, about 9,000 patients are treated for a hip fracture each year (Gjertsen et al. 2008). A high proportion of hip fracture patients have cognitive impairment (Mundi et al. 2014, Mukka et al. 2017, Kristoffersen et al. 2019). Cognitive impairment is defined as a decrease in cognition beyond normal aging (Hugo and Ganguli 2014). It can be mild, it can include dementia, or it might be temporary such as in delirium (Petersen et al. 2001, Holsinger et al. 2007). Dementia is usually diagnosed according to ICD-10 criteria in Norway (Naik and Nygaard 2008), and is dependent on a history of cognitive impairment of at least 6 months’ duration in activities of daily living.

Despite high prevalence of cognitive impairment among hip fracture patients, these patients are often excluded from research (Mundi et al. 2014).

We investigated whether the presence of cognitive impairment affects the choice of surgical treatment for different types of hip fractures, and evaluated whether patients with cognitive impairment have a different risk of reoperation and mortality compared with cognitively fit patients.

## Patients and methods

### Study design

This is a prospective observational study based on data from the Norwegian Hip Fracture Register (NHFR).

The NHFR collects data from all hospitals in Norway treating hip fractures (Gjertsen et al. 2008). Data are reported by the surgeon on a 1-page form with information on the fracture type, the operation method, and the patient, including assessment of cognitive impairment. Femoral neck fractures are classified according to the Garden classification. Trochanteric fractures are classified according to the AO/OTA classification.

The surgeon evaluates patients’ cognitive function by examining their medical chart, asking them or their relatives, or using the Clock Drawing Test (Amodeo et al. 2015). Since the form is completed immediately after the operation, the information on cognitive function must be collected preoperatively. The NHFR has no data on the methods the surgeons used to obtain information on cognitive function. The question concerning cognitive impairment on the form is: “Does the patient have cognitive impairment?” Surgeons answer “Yes,” “No,” or “Uncertain.” The data on cognitive impairment reported to the NHFR have been validated against external quality databases. The positive predictive value of the data reported to the NHFR on cognitive impairment was 78% (Kristoffersen et al. 2019).

The completeness of reporting of primary hip fracture operations to the NHFR has been found to be 88% for osteosynthesis and 94% for hemiarthroplasty when compared with the Norwegian Patient Register (Furnes et al. 2017).

Reoperations are linked to the primary operation by the unique identification number assigned to each inhabitant in Norway. Total hip arthroplasty revisions are reported on separate operation forms to the Norwegian Arthroplasty Register and later duplicated to the files of the NHFR.

It is possible to report several reasons for each reoperation, and a hierarchy of reasons was drawn up. If a deep or superficial infection was present, this was defined as the main reason for reoperation.

### Patient selection

**Figure 1.**
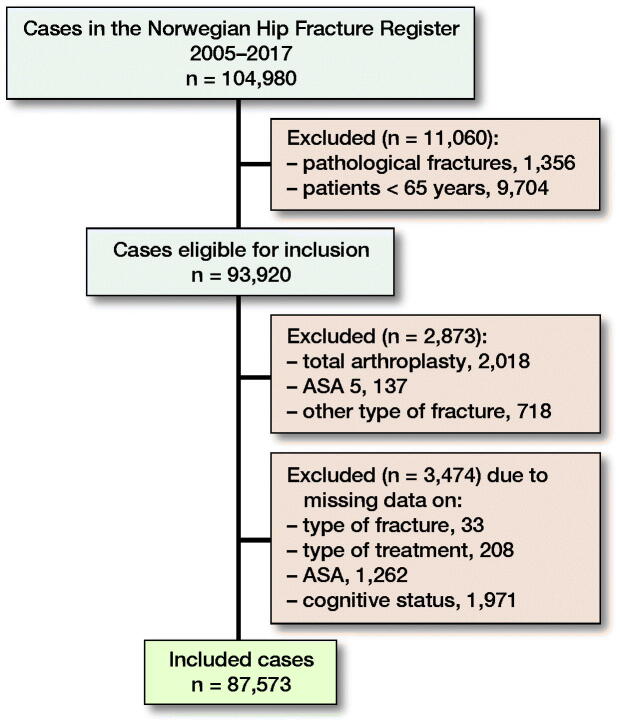
Flowchart.

In the period 2005–2017, 104,980 primary hip fracture operations were reported to the NHFR. For the present study, pathological fractures and fractures in patients younger than 65 years of age were excluded (n = 11,060). Total hip arthroplasty for hip fracture was also excluded, since these operations are reported on separate forms to the Norwegian Arthroplasty Register with no information on cognitive function (n = 2,018). Further, fractures in ASA 5 patients, other fracture types than femoral neck, trochanteric or subtrochanteric fractures, operations with missing data on type of fracture, type of surgery, ASA classification, and cognitive status were excluded (n = 4,329) (Figure 1). Finally, 87,573 operations were included in the analysis.

### Statistics

The patients were analyzed in groups according to their cognitive function: cognitively impaired, cognitively fit, and uncertain cognitive function (where the surgeon was uncertain of the patient’s cognitive function). Pearson’s chi-square test was used to compare categorical variables. Independent samples t-tests and analyses of variance (ANOVA), were used to compare the means for continuous variables. P-values < 0.05 were considered statistically significant. The Kaplan–Meier method was used to calculate time from primary surgery to reoperation. Hazard rate ratios (HRRs) are presented with 95% confidence intervals (CIs). Differences in reoperation risks between the groups were calculated using a Cox regression model with adjustments for sex, age, ASA class, fracture type, and operation method. Separate analyses were conducted for reoperations after primary osteosynthesis and those following hemiarthroplasty. Sub-analyses were performed for reoperations after hemiarthroplasty by surgical approach and fixation method. Further, the Cox regression model was used to analyze differences in mortality between the different patient groups with patients with no cognitive impairment as reference. 30-day, 90-day, and 1-year mortality were calculated with adjustments for sex, age, ASA, fracture type, and operation method. The proportional hazards assumption was fulfilled when investigated visually using log-minus-log plots. Fine and Gray analysis was also used to determine whether mortality was a competing risk in reoperation.

The statistical software package IBM SPSS Statistics, version 24.0 (IBM Corp, Armonk, NY, USA) and the statistical package R, version 3.6.0 (R Foundation for Statistical Computing, Vienna, Austria) were used for the statistical analysis. The study was performed in accordance with the REporting of studies Conducted using Observational Routinely-collected health Data (RECORD) statement (Benchimol et al. 2015).

### Ethics, funding, and potential conflict of interest

The NHFR has permission from the Norwegian Data Protection Authority to collect and store data on hip fracture patients (permission issued January 3, 2005; reference number 2004/1658-2 SVE/-). The patients signed a written, informed consent declaration, and when unable to understand or sign, their next of kin could sign the consent form on their behalf. The Norwegian Hip Fracture Register is financed by the Western Norway Regional Health Authority. No competing interests were declared.

## Results

In the 87,573 hip fracture operations, 27% of the patients had been classified by the surgeon as cognitively impaired and 63% as cognitively fit. In 10% of the operations the surgeon had evaluated the patient’s cognitive function as “uncertain.” The mean follow-up time was 3.0 years (3.0–3.0). Patients with cognitive impairment had a mean follow-up time of 1.8 years (1.8–1.9), non-impaired patients 3.6 years (3.5–3.6) and “uncertain” patients 2.5 years (2.5–2.6).

### Baseline data

There were 72% women among the patients. The patients with cognitive impairment were on average 3.5 years older and had more severe comorbidity (higher ASA score) than non-impaired patients (Table 1).

**Table 1.** Baseline data for patients by cognitive function. Values are frequency (%) unless otherwise specified

Factor	Total	No	Cognitive impairment Uncertain	Yes
Total	87,573	54,859 (63)	8,985 (10)	23,729 (27)
Women	62,751 (72)	39,182 (71)	6,332 (71)	17,237 (73)
Mean age (SD)	83.2 (7.5)	82.0 (7.8)	84.8 (7.0)	85.5 (6.4)
Age group				
65–74	12,611 (14)	10,388 (19)	793 (8.8)	1,430 (6.0)
75–79	12,837 (15)	9,120 (17)	1,099 (12)	2,618 (11)
80–84	20,309 (23)	12,727 (23)	2,028 (23)	5,554 (23)
85–89	23,494 (27)	13,247 (24)	2,754 (31)	7,493 (32)
≥ 90	18,322 (21)	9,377 (17)	2,311 (26)	6,634 (28)
ASA class				
ASA 1 + 2	32,293 (37)	24,298 (44)	2,485 (28)	5,510 (23)
ASA 3 + 4	55,280 (63)	30,561 (56)	6,500 (72)	18,219 (77)
Fracture type				
Undisplaced FNF	12,782 (15)	8,166 (15)	1,223 (14)	3,393 (14)
Displaced FNF	37,006 (42)	22,978 (42)	3,780 (42)	10,248 (43)
Basocervical FNF	3,112 (3.6)	1,918 (3.5)	328 (3.7)	866 (3.6)
Trochanteric A1 **^a^**	14,768 (17)	9,168 (17)	1,549 (17)	4,051 (17)
Trochanteric A2 **^a^**	14,012 (16)	8,743 (16)	1,512 (17)	3,757 (16)
Trochanteric A3 **^a^**	1,439 (1.6)	931 (1.7)	143 (1.6)	365 (1.5)
Subtrochanteric	4,454 (5.1)	2,955 (5.4)	450 (5.0)	1,049 (4.4)
Primary operation				
Screw osteosynthesis	16,938 (19)	10,483 (19)	1,707 (19)	4,748 (20)
Hemiarthroplasty	32,667 (37)	20,522 (37)	3,284 (37)	8,861 (37)
Sliding hip screw	27,161 (31)	16,956 (31)	2,827 (31)	7,378 (31)
Short IM nail	7,265 (8.3)	4,529 (8.3)	815 (9.1)	1,921 (8.1)
Long IM nail	3,542 (4.0)	2,369 (4.3)	352 (3.9)	821 (3.5)
Surgical approach				
Anterior/anterolateral	2,495 (7.6)	1,604 (7.8)	254 (7.7)	637 (7.2)
Lateral	26,401 (81)	16,596 (81)	2,680 (82)	7,125 (80)
Posterior	3,286 (10)	2,008 (9,8)	308 (9.4)	970 (11)
Other/missing data	485 (1.5)	314 (1.5)	42 (1.3)	129 (1.4)
Fixation of HA				
Cemented	24,278 (74)	15,353 (75)	2,408 (73)	6,517 (74)
Uncemented	7,851 (24)	4,854 (24)	804 (25)	2,193 (25)
Missing data	538 (1.6)	315 (1.5)	72 (2.2)	151 (1.7)

FNF = femoral neck fracture, IM = intramedullary, HA = hemiarthroplasty.

a AO/OTA classification.

Displaced femoral neck fractures (FNFs) constituted 42% of all fractures. Only small differences in the distribution of fractures and operation methods were found between the groups but, due to the large numbers, some of these small differences were statistically significant (Table 1).

Surgical methods for each fracture type were not influenced by the patients’ cognitive function (Figure 2, see Supplementary data). The most common operation methods were hemiarthroplasty (37%) and osteosynthesis with a sliding hip screw (31%) (Table 1). Most hemiarthroplasties were performed with a lateral approach (81%) and three-quarters of hemiarthroplasties were cemented (Table 1).

### Reoperations

Cox regression analysis and the Fine and Grey method showed a similar risk of reoperation (Ranstam and Robertsson 2017) (Table 2).

**Table 2.** Number of reoperations and risk of reoperation after hip fracture surgery by cognitive function using Cox regression model and Fine and Gray model with adjustments for age, sex, ASA classification, fracture type, and treatment

Cognitive impairment	Total n	Reoperation n (%)	Cox regression Hazard Rate ratio (95% CI)	Fine and Gray Hazard Rate ratio (95% CI)
Total	87,573	6,568 (7.5)		
No	54,859	4,860 (8.9)	1 Reference	1 Reference
Uncertain	8,985	598 (6.7)	0.91 (0.83–0.99)	0.91 (0.84–0.99)
Yes	23,729	1,110 (4.7)	0.71 (0.66–0.76)	0.69 (0.65–0.74)
Hemiarthroplasty	32,667	1,425 (4.4)		
No	20,522	873 (4.3)	1 Reference	1 Reference
Uncertain	3,284	169 (5.1)	1.3 (1.1–1.6)	1.3 (1.1–1.6)
Yes	8,861	383 (4.3)	1.2 (1.1–1.4)	1.2 (1.0–1.3)
Osteosynthesis	54,906	5,143 (9.4)		
No	34,337	3,987 (11)	1 Reference	1 Reference
Uncertain	5,701	429 (7.5)	0.81 (0.73–0.89)	0.85 (0.77–0.94)
Yes	14,868	727 (4.9)	0.58 (0.53–0.63)	0.62 (0.57–0.67)

The overall reoperation rate for all patients was 7.5% (n = 6,568) (Table 2). Patients with cognitive impairment had an overall reoperation rate of 4.7%, compared with 8.9% for cognitively fit patients (HRR 0.71; CI 0.66–0.76). Patients with “uncertain” cognitive function had a reoperation rate of 6.7% (HRR 0.91; CI 0.83–0.99).

The overall reoperation rates for all patients were 4.4% after hemiarthroplasty and 9.4% after osteosynthesis. The reoperation risk for patients with cognitive impairment was slightly higher for hemiarthroplasty (HRR 1.2; CI 1.1–1.4) but lower for osteosynthesis (HRR 0.58; CI 0.53–0.63) than for those without cognitive impairment (Table 2).

There were small differences in risk of reoperation between patients with and without cognitive impairment for those operated with hemiarthroplasty due to infection and periprosthetic fracture.

Analysis by fixation of the hemiprosthesis showed that patients with cognitive impairment treated with uncemented hemiarthroplasty had a higher risk of reoperation for any reason (HRR 1.3; CI 1.1–1.7) and a particularly high risk due to periprosthetic fracture (HRR 1.6; CI 1.0–2.6), compared with patients without cognitive impairment. No such differences could be found for cemented hemiarthroplasty. Further, cognitively impaired patients treated with hemiarthroplasty had a higher risk of reoperation because of dislocation than non-impaired patients (1.5% vs. 1.0%, HRR 1.7; CI 1.3–2.1) (Table 3). Analysis by surgical approach showed that this risk was higher with the posterior approach (4.7% vs. 2.8%, HRR 1.8; CI 1.2–2.7) and lower with the lateral approach (1.1% vs. 0.8%, HRR 1.5; CI 1.1–2.0).

**Table 3.** Reasons for reoperation after hemiarthroplasty and osteosynthesis. Reoperations appear in the order of our hierarchy. Values are frequency (%)

Factor	Total	No	Cognitive impairment Uncertain	Yes
All reoperations	6,568 (7.5)	4,860 (8.9)	598 (6.7)	1,110 (4.7)
Reoperation after hemiarthroplasty	1,425 (4.4)	873 (4.4)	169 (5.1)	383 (4.3)
Infection	672 (2.1)	416 (2.0)	81 (2.5)	175 (2.0)
Periprosthetic fracture	151 (0.5)	90 (0.4)	17 (0.5)	44 (0.5)
Dislocation of prosthesis	395 (1.2)	206 (1.0)	55 (1.7)	134 (1.5)
Loosening of hemiarthroplasty	18 (0.1)	17 (0.1)	0 (0.0)	1 (0.0)
Sequelae of femoral neck fracture **^a^**	31 (0.1)	24 (0.1)	2 (0.1)	5 (0.1)
Other reason	158 (0.5)	120 (0.5)	14 (0.4)	24 (0.3)
Reoperation after osteosynthesis	5,143 (9.4)	3,987 (12)	429 (7.5)	727 (4.9)
Infection	225 (0.4)	136 (0.4)	29 (0.5)	60 (0.4)
Peri-implant fracture	363 (0.7)	247 (0.7)	34 (0.6)	82 (0.6)
Avascular necrosis	346 (0.6)	248 (0.7)	29 (0.5)	69 (0.5)
Osteosynthesis failure	1,541 (2.8)	1022 (3.0)	172 (3.0)	320 (2.2)
Cut-out	142 (0.3)	107 (0.3)	12 (0.2)	23 (0.2)
Non-union	276 (0.5)	212 (0.6)	27 (0.5)	37 (0.2)
Sequelae of proximal femoral fracture **^a^**	1,744 (3.2)	1,568 (4.6)	96 (1.7)	80 (0.5)
Local pain due to osteosynthesis material	360 (0.7)	318 (0.9)	15 (0.3)	27 (0.2)
Other reason	173 (0.3)	129 (0.4)	15 (0.3)	29 (0.2)

a Reoperation with total hip arthroplasty reported to the Norwegian Arthroplasty Register.

Few patients with cognitive impairment were reoperated due to osteosynthesis failure and local pain (Table 3). Only 0.5% of cognitively impaired patients treated with osteosynthesis had revision total hip arthroplasty, compared with 4.6% of cognitively fit patients.

### Mortality

30-day mortality was 13% for cognitively impaired patients and 4.6% for cognitively fit patients (HRR 2.2; CI 2.1–2.3). 90-day mortality was 23% for cognitively impaired patients and 8.5% for cognitively fit patients (HRR 2.2; CI 2.1–2.3). Finally, 1-year mortality was 38% for cognitively impaired patients and 16% for cognitively fit patients (HRR 2.1; CI: 2.1–2.2) (Table 4, see Supplementary data). Patients with cognitive impairment had a greater overall mortality risk than cognitively fit patients (HRR 2.1; CI 2.0–2.1).

## Discussion

There was no difference in type of fracture or type of initial treatment among hip fracture patients in relation to cognitive function in NHFR. This supports the idea of equal treatment for all hip fracture patients. The lower reoperation rate for patients with cognitive impairment found in our study does not necessarily imply that these patients do better than those without cognitive impairment.

Patients with cognitive impairment have been reported to have a higher risk of poorer functional outcome after hip fracture incidents (Sheehan et al. 2018). Hip fracture patients with cognitive impairment are older and have comorbidities that increase the risk of any reoperation. It is easier for cognitively fit patients to tolerate the peri- and postoperative strain and stress of revision surgery. Patients with cognitive impairment might not be offered surgical revision due to a higher risk of complications such as prosthesis dislocation and shorter life expectancy than in non-impaired patients.

An infection is probably the most feared complication after hip fracture surgery. In most cases, an infection leaves no other options than surgical debridement. Notably, cognitive impairment, in our study, did not seem to increase the risk of reoperation due to infection. Cognitively impaired patients treated with hemiarthroplasty had an increased risk of prosthesis dislocation, especially when the posterior approach had been used. Our results concur with those in the study by Svenøy et al. (2017), who reported an 8-fold increase in risk of dislocation after the posterior approach compared with the lateral. Our results suggest that the use of the posterior approach in cognitively impaired patients should be avoided.

It is well established that uncemented hemiarthroplasties have a higher risk of revision than cemented (Langslet et al. 2014, Kristensen et al. 2020).

In our study, cognitively impaired patients treated with uncemented hemiarthroplasty had a higher risk of reoperation for any reason and for periprosthetic fracture than non-impaired patients. No such differences were found for cemented hemiarthroplasties. Thus, uncemented hemiarthroplasties seem to yield inferior results and should not be used in cognitively impaired patients who may have a particularly high risk of recurrent falls and periprosthetic fracture.

Very few patients with cognitive impairment were reoperated with a total hip arthroplasty, which may be contraindicated in these patients because of lack of compliance and increased risk of dislocation. However, the risk of dislocation can be reduced with the use of a dual-mobility cup (Jobory et al. 2019).

Our study also included patients where the orthopedic surgeon had been in doubt whether the patient had cognitive impairment or not. These patients performed as an intermediate group in our analysis. One explanation could be that these patients may have had delirium, which is common in patients with hip fracture and complicates the assessment of chronic cognitive impairment and dementia. Delirium is also a risk factor for developing dementia after a hip fracture (Krogseth et al. 2011).

Mortality increased 2-fold for patients with cognitive impairment, both from 30 to 90 days and from 90 days to 1 year. This finding is in line with previous studies (Söderqvist et al. 2006, Mukka et al. 2017). Our study does not include information on causes of mortality. Holvik et al. (2010) found that predictors of mortality in older hip fracture patients were admission from a nursing home, comorbidity, and frailty. All these predictors are associated with cognitively impaired patients.

We have not analyzed patient-reported outcomes, and therefore have no information on how the hip fractures influenced the patients’ quality of life and how the patients performed who were not reoperated.

### Strengths and limitations

The large number of patients in our study is an advantage and enabled us to analyze rare complications and causes of reoperation. One should, however, be careful to draw conclusions based on very small differences even if they reach statistical significance. One important limitation of the study is the accuracy of the surgeon’s assessment of cognitive function. An earlier study from the NHFR found that orthopedic surgeons identified cognitive impairment with a specificity of 90%, a sensitivity of 69%, positive predictive value of 78%, and negative predictive value of 84%, compared with information recorded in local hospital databases (Kristoffersen et al. 2019).

The completeness of the reported reoperations has been found to be lower than the reporting of primary hip fracture operations in the NHFR when compared with the Norwegian Patient Register (Furnes et al. 2017). We have, however, no indication that the reporting of reoperations differs between the patient groups according to cognitive function. Accordingly, the hazard rate ratios in this study are probably reliable, but the crude number of reoperations may represent a best-case scenario and the actual number of reoperations may be higher. Follow-up time and mortality differed between the treatment groups. Many of the causes of reoperations, such as pain and loosening of the implant, may occur a long time after primary surgery. When comparing the treatment groups, one should therefore be aware that patients with cognitive impairment might die before the complications occur.

## Conclusion

The results suggest that patients with cognitive impairment should be treated with the same surgical procedures as patients without cognitive impairment. However, hemiarthroplasty with uncemented stem and a posterior approach should probably be avoided in cognitively impaired patients due to the increased risk of periprosthetic fracture and dislocation.

## Supplementary data

Figure 2 and Table 4 are available as supplementary data in the online version of this article, http://dx.doi.org/10.1080/17453674.2019.1709712

## Supplementary Material

Supplemental Material
