# Supplementary material for: Cognitive impairment influences the risk of reoperation after hip fracture surgery: results of 87,573 operations reported to the Norwegian Hip Fracture Register
Source: Acta Orthop. 2020 Jan 13;91(2):146–51. doi: 10.1080/17453674.2019.1709712 (PMC7144309; doi:10.1080/17453674.2019.1709712)
Supplement: Supplemental Material [file IORT_A_1709712_SM2156.pdf]

Supplementary data

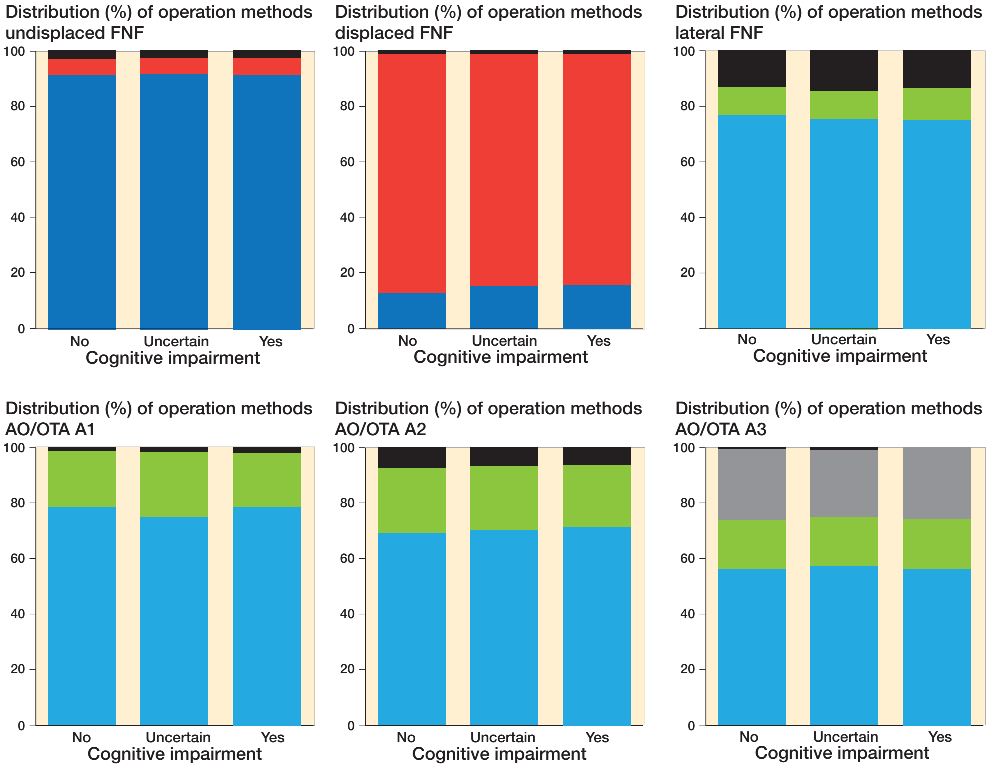

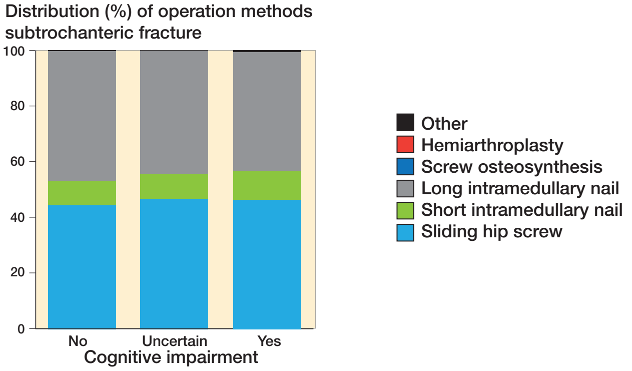

Figure 2. Distribution of primary operation methods by cognitive function for different fractures.
FNF = femoral neck fracture.
AO/OTA A1 = two-part trochanteric fractures.
AO/OTA A2 = multi-fragment trochanteric fractures.
AO/OTA A3 = intertrochanteric femoral fractures.

Table 4. Death and mortality risk after hip fracture surgery by cognitive function. Cox regression with adjustments for age, sex, ASA classification, fracture type, and treatment

Mortality<br>Cognitive impairment	Total<br>n	Dead<br>n (%)	Hazard Rate<br>ratio (95% CI)
Overall mortality	87,573	51,164 (58)	
No	54,859	27,307 (50)	1 reference
Uncertain	8,985	6,361 (71)	1.6 (1.5–1.6)
Yes	23,729	17,496 (74)	2.1 (2.0–2.1)
30-day mortality	87,573	6,452 (7.4)	
No	54,859	2,507 (4.6)	1 reference
Uncertain	8,985	865 (9.6)	1.6 (1.5–1.8)
Yes	23,729	3,080 (13)	2.2 (2.1–2.3)
90-day mortality	87,573	11,652 (13)	
No	54,859	4,676 (8.5)	1 reference
Uncertain	8,985	1,547 (17)	1.6 (1.6–1.7)
Yes	23,729	5,429 (23)	2.2 (2.1–2.3)
1-year mortality	87,573	20,511 (23)	
No	54,859	8,851 (16)	1 reference
Uncertain	8,985	2,687 (30)	1.6 (1.5–1.7)
Yes	23,729	8,973 (38)	2.1 (2.1–2.2)
